# Integrative pan-cancer analysis reveals the prognostic and immunological significance of SLC7 family cationic amino acid transporters

**DOI:** 10.1038/s41598-025-34723-8

**Published:** 2026-01-08

**Authors:** Gang Peng, Hao Peng, Zhe Shao, Liangqi Jiang, Mingrui Li, Yang Li

**Affiliations:** 1 Department of Neurosurgery, Xiangya Hospital, Central South University, 87 Xiangya Road, Changsha, 410008 Hunan P.R. China; 2 Department of Neurosurgery, Hainan Affifiliated Hospital of Hainan Medical University, 19 Xiuhua Road, Haikou, 570311 Hainan P.R. China; 3 Department of Neurosurgery, The second People’s Hospital of Hainan Province, 27Aoya Road, Wuzhishan, 572299 Hainan P.R. China

**Keywords:** SLC7 family, Pan-cancer, Tumor immune microenvironment, Amino acid, Computational biology and bioinformatics, Computational models, Genome informatics, Cancer, Cancer metabolism, Cancer therapy, CNS cancer

## Abstract

Amino acid metabolism plays a crucial role in cancer progression and invasion by modulating immune responses within the tumor microenvironment and tumor immune response. Transmembrane transport of cellular amino acids is mediated by members of the cationic amino acid transporters SLC7 family, a group of cationic amino acid transporters. Dysregulation of SLC7 family genes can disrupt systemic homeostasis, contributing to a variety of diseases, including multiple cancer types. Additionally, SLC7 family members can alter amino acid metabolic plasticity, further influencing tumor adaptation and progression. However, the precise impact of SLC7 family genes within the cancer microenvironment remains largely unexplored. This study analyzed the expression patterns, genomic alterations (single nucleotide variants, copy number variations, methylation levels), pathway activations, and clinical and subtype associations of SLC7 family genes across The Cancer Genome Atlas (TCGA) pan-cancer cohort. SLC7 activity was quantified using the ssGSEA function of the “GSVA” package to generate the SLC7 score, which was found to correlate with oncogenic and immune-related pathways, including mTORC1 signaling, interferon-gamma response, and inflammatory pathways. In vitro validation was conducted using multiple cancer cell lines (A549, H1299, HCT116, FaDu, Panc-1) and counterparts (BxPC-3, SW480, H460, BEAS-2B, FHC, HaCaT). Gene knockdown was achieved by siRNA transfection, followed by quantitative PCR, Western blotting, and functional assays assessing proliferation, invasion, and ferroptosis (iron and GSH levels) under erastin treatment. SLC7 family genes showed significant associations with immune infiltration and poor prognosis across various cancers. The SLC7 score demonstrated strong positive correlations with malignant and immune-related pathways, such as mTORC1 signaling, interferon-gamma response, and inflammatory response across the pan-cancer cohort. Elevated SLC7 scores were also associated with enhanced immune-related characteristics, and SLC7 genes, particularly SLC7A8 and SLC7A9, emerged as promising predictive markers for response to immune checkpoint inhibitor therapy (Atezolizumab) in cancer patients. Among SLC7 family genes, SLC7A5 expression was markedly elevated in KRAS-mutant tumor cell lines and was required for KRAS-driven proliferation and invasion. SLC7A11 was highly expressed in multiple tumor types and protected cancer cells from ferroptosis by maintaining intracellular cystine uptake and redox homeostasis. Individuals exhibiting elevated SLC7 scores and aberrant expression of SLC7 family genes demonstrate significant immune-inflammatory tumor immune microenvironment (TIME) characteristics and predictive potential for immunotherapy. Thus, the SLC7 family genes have the potential to serve as valuable biomarkers and therapeutic targets for cancer patients.

**Supplementary Information:**

The online version contains supplementary material available at 10.1038/s41598-025-34723-8.

## Introduction

Amino acid metabolism has emerged as a pivotal regulatory component in cancer-associated pathways within cell biology. Alterations, including the absence, overexpression, and dysfunction of specific amino acid transporters, are increasingly linked to tumorigenesis and the progression of solid tumors^1^. The solute carrier family 7 (SLC7) subfamily, which includes both cationic amino acid transporters (CATs) such as SLC7A1, SLC7A2, SLC7A3, SLC7A4, and SLC7A14, as well as the L-type amino acid transporters (LATs) including SLC7A5 and SLC7A15, serves crucial roles in normal cellular function by transporting essential amino acids^2,3^. Specifically, CATs function as facilitated diffusers, enabling the primary entry of cationic amino acids into cells^2^, while LATs, also known as glycoprotein-related amino acid transporters, are essential for handling large amino acids(AAs)^4^. Despite their importance in cellular homeostasis, the role of the SLC7 family as potential therapeutic targets in cancer remains largely unexplored.

Previous studies suggest that SLC7 family genes are implicated in cancer development, progression, invasion, and drug resistance. For example, elevated SLC7A1 expression correlates with poor prognosis in ovarian cancer^5^, while high SLC7A2 expression inhibits cellular viability, invasion, and migration in ovarian cancer cells^6^. SLC7A2 downregulation in hepatocellular carcinoma leads to increased CXCL1 expression via the PI3K/Akt/NF-κB pathway^7^. Additionally, SLC7A3, activated by p53, facilitates adaptation to glutamine deprivation^8^, and SLC7A5 is essential for the growth of KRAS-mutant colorectal cancer^9^. Other SLC7 family members, including SLC7A4^10^, SLC7A7^11–14^, SLC7A8^15,16^, SLC7A9^16^, SLC7A11^17–19^, have also been linked to cancer progression, indicating their potential as therapeutic targets. However, a comprehensive understanding of the SLC7 family’s mechanistic role in tumor immunity across various cancers remains lacking.

The tumor microenvironment (TME) encompasses not only cancer cells but also a complex ecosystem, including immune cells, extracellular matrix proteins, and metabolites that support tumor survival and immune evasion^20,21^. Within the TME, immune responses are highly dependent on the acquisition of extracellular nutrients, particularly amino acids^22^. SLC7 transporters play critical roles in T cell functions—such as activation, differentiation, homeostasis, and memory^23^—with specific members, like SLC7A5 and sodium-coupled neutral amino acid transporters, upregulated in activated T cells^24–26^. For instance, SLC7A5 is essential for CD8 + T cell responses, forming a heterodimer with CD98 that is involved in T cell activation, differentiation, memory formation, and in regulating Th1 and Th17 cells^24^. Additionally, SLC7A1-4 facilitates arginine transport, fueling diverse downstream metabolic pathways with multifaceted immune functions^27^. These findings underscore the essential role of SLC7 family members in T cell activation and innate immune responses, yet their specific roles within tumor immunity are not fully understood.

In this study, we conducted a systematic analysis of SLC7 family gene landscapes across 33 cancer types, examining genomic alterations (single nucleotide variants, copy number variations, methylation levels), mRNA expression, pathway activity based on protein levels, and prognostic significance. An SLC7 score was calculated to assess its associations with prognosis, clinical features, subtype characteristics, immune cell infiltration in tumors, drug sensitivity predictions, and responses to immunotherapy, providing insights into the therapeutic potential of the SLC7 family in oncology.

## Materials and methods

### Data acquisition

Expression profiles (RSEM mRNA expression) and clinical data from The Cancer Genome Atlas (TCGA) were obtained through the UCSC Xena database (https://xenabrowser.net/datapages/). The Human Protein Atlas (https://www.proteinatlas.org/) was used to assess protein expression of SLC7 family genes via immunohistochemistry. Pathway activity scores were calculated using Reverse Phase Protein Array (RPPA) data from The Cancer Proteome Atlas (TCPA) database (https://tcpaportal.org/tcpa/). Immune-related data, including immune cell populations, immune scores, and immune gene sets, were retrieved from the ImmuCellAI (http://bioinfo.life.hust.edu.cn/ImmuCellAI#!/), ESTIMATE (https://bioinformatics.mdanderson.org/estimate/), and the ‘IOBR’ R package^28^. Additionally, IC50 data for small molecules in cell lines and corresponding mRNA expression profiles were downloaded from the Cancer Therapeutics Response Portal (CTRP, http://portals.broadinstitute.org/ctrp/) and Genomics of Drug Sensitivity in Cancer (GDSC, https://www.cancerrxgene.org/). The immunotherapy dataset was extracted from the ‘IMvigor210CoreBiologies’ package^29^.

### Online analysis

Data on genomic and epigenetic alterations (including single nucleotide variants, copy number variations, and methylation levels), pathway activities, and drug sensitivities from the GDSC and CTRP databases were obtained through the Gene Set Cancer Analysis (GSCA) platform (http://bioinfo.life.hust.edu.cn/GSCA/#/), which integrates genomic and drug sensitivity data for gene set cancer analysis^30^. Details of the gene sets used in this study can be directly accessed from the GSVA database.

SLC7 Score Calculation and Enrichment Analysis.

The SLC7 score, representing the integrated expression level of the SLC7 gene family, was computed for each sample using the GSVA algorithm. This score reflects the average expression of the SLC7 family across the pan-cancer cohort. Correlations between the SLC7 score and pathway activity scores for HALLMARK pathways were assessed using Pearson correlation analysis; pathway data was sourced from the MSigDB database (http://www.gsea-msigdb.org/, project: c2.cp.v7.5.1.symbols.gmt). The GSVA algorithm was similarly applied to calculate pathway activity scores.

### Prognostic analysis

To assess the prognostic impact of the SLC7 score and the SLC7 gene family on survival, univariate Cox regression analyses were conducted across the pan-cancer cohort using the ‘survimine’ R package.

Evaluation of Tumor Immune Microenvironment (TIME).

The ESTIMATE algorithm was used to calculate stromal, immune, and tumor purity scores for each patient. SLC7 family gene data and SLC7 scores were accessed from the GSCA platform, while immune gene lists were obtained from the IOBR package. Associations among the SLC7 score, immune infiltration levels, and immune-related gene expression (including modulatory, MHC, and chemokine or chemokine receptor genes) were analyzed within the pan-cancer cohort. Visualizations, including heatmaps and bubble plots, were generated using the ‘ggplot2’ R package.

### Cell culture and transfection

All tumor cell lines (A549, H1299, HCT116, FaDu, BxPC-3, Panc-1, SW480, H460) and normal epithelial cell lines (BEAS-2B, FHC, HaCaT) were obtained from the Cell Bank of the Shanghai Institutes of Biological Sciences (Shanghai, China). Cells were cultured in Dulbecco’s modified Eagle’s medium (DMEM; HyClone, United States) supplemented with 10% fetal bovine serum (FBS) and maintained at 37 °C in a humidified atmosphere containing 5% CO₂. Small interfering RNAs (siRNAs) targeting SLC7A11 and a non-targeting negative control (siNC) were synthesized by GenPharma (Suzhou, China). Transfections were performed using Lipofectamine^®^ RNAiMAX (Invitrogen, Carlsbad, CA, United States) according to the manufacturer’s protocol. Three siRNA sequences (si-1, si-2, si-3) were designed to target exon 1–3 of SLC7A11, and the two most effective siRNAs (si-1 and si-2) were used for subsequent experiments.

### RNA extraction and quantitative real-time PCR (qRT-PCR)

Total RNA was extracted from cells using TRIzol reagent (Invitrogen, Carlsbad, CA, United States). Complementary DNA (cDNA) was synthesized using the PrimeScript™ RT Reagent Kit (Takara, Dalian, China), and qRT-PCR was performed using SYBR^®^ Premix Ex Taq™ (Takara, Dalian, China) on a QuantStudio™ 6 Flex Real-Time PCR System (Applied Biosystems, USA). β-Actin was used as an internal control. The relative expression of target genes (SLC7A11, GPX4, and Fis1) was calculated using the 2^–ΔΔCt method.

### Cell proliferation and migration assay

Cell proliferation was assessed using the Cell Counting Kit-8 (Sigma-Aldrich, Shanghai, China) following the manufacturer’s instructions. Tumor cells including KRAS-mutant (Panc-1, HCT116, A549) and KRAS-wildtype (BxPC-3, SW480, H460), were seeded in 96-well plates at a density of 5000 cells per well in triplicate. After incubation for 24, 48, 72, and 96 h, 10 µL of CCK-8 reagent was added to each well and incubated for an additional 2 h at 37 °C. Absorbance was measured at 450 nm using a microplate reader (Bio-Rad, USA). Cell growth curves were plotted based on optical density (OD) values at each time point. Cells (5 × 10⁴) suspended in 200 µL of serum-free medium were added to the upper chamber, while 600 µL of medium containing 10% FBS was added to the lower chamber as a chemoattractant. The cell migration assay was performed using 24-well transwell chambers (Corning, NY, United States) according to the manufacturer’s instructions. All assays were performed in triplicate.

### Measurement of intracellular iron and GSH levels

After transfection with siRNAs for 48 h, cells were treated with DMSO or the ferroptosis inducer erastin (10 µM, Selleck Chemicals, USA) for 24 h. Cellular ferrous iron (Fe²⁺) content was determined using an Iron Assay Kit (Abcam, Cambridge, UK), and intracellular glutathione (GSH) levels were measured using a GSH Assay Kit (Abcam, Cambridge, UK) according to the manufacturer’s instructions. The absorbance values were normalized to total protein content, and results were expressed as nmol/mg protein.

### Western blot analysis

Cells were lysed in 300 µL SDS sample buffer (Sangon Biotech, Shanghai, China) supplemented with 1 mM PMSF and 1 mM phosphatase inhibitor. Protein concentrations were determined using a BCA Protein Assay Kit (Beyotime, China). Equal amounts of protein (20 µg) were separated by 10–15% SDS-PAGE and transferred onto PVDF membranes (Millipore, USA). After blocking with 5% non-fat milk for 1 h at room temperature, membranes were incubated with primary antibodies against SLC7A11, SLC7A5, GPX4, or α-Tubulin/GAPDH (Cell Signaling Technology, USA) overnight at 4 °C, followed by HRP-conjugated secondary antibodies for 2 h at room temperature. Protein bands were visualized using ECL reagents (Sangon Biotech, China).

#### Statistical analysis

Group comparisons were conducted using Student’s t-test, Mann–Whitney U test, or Games-Howell test. Box plot analyses were performed with the ‘ggplot2’, ‘ggpubr’, and ‘ggstatplot’ R packages. Pearson and Spearman correlation coefficients were used in correlation analyses. Statistical significance was defined as a p-value < 0.05.

## Result

### Upregulation of the SLC7 family correlates with poor prognosis in multiple cancers

**Fig. 1:**
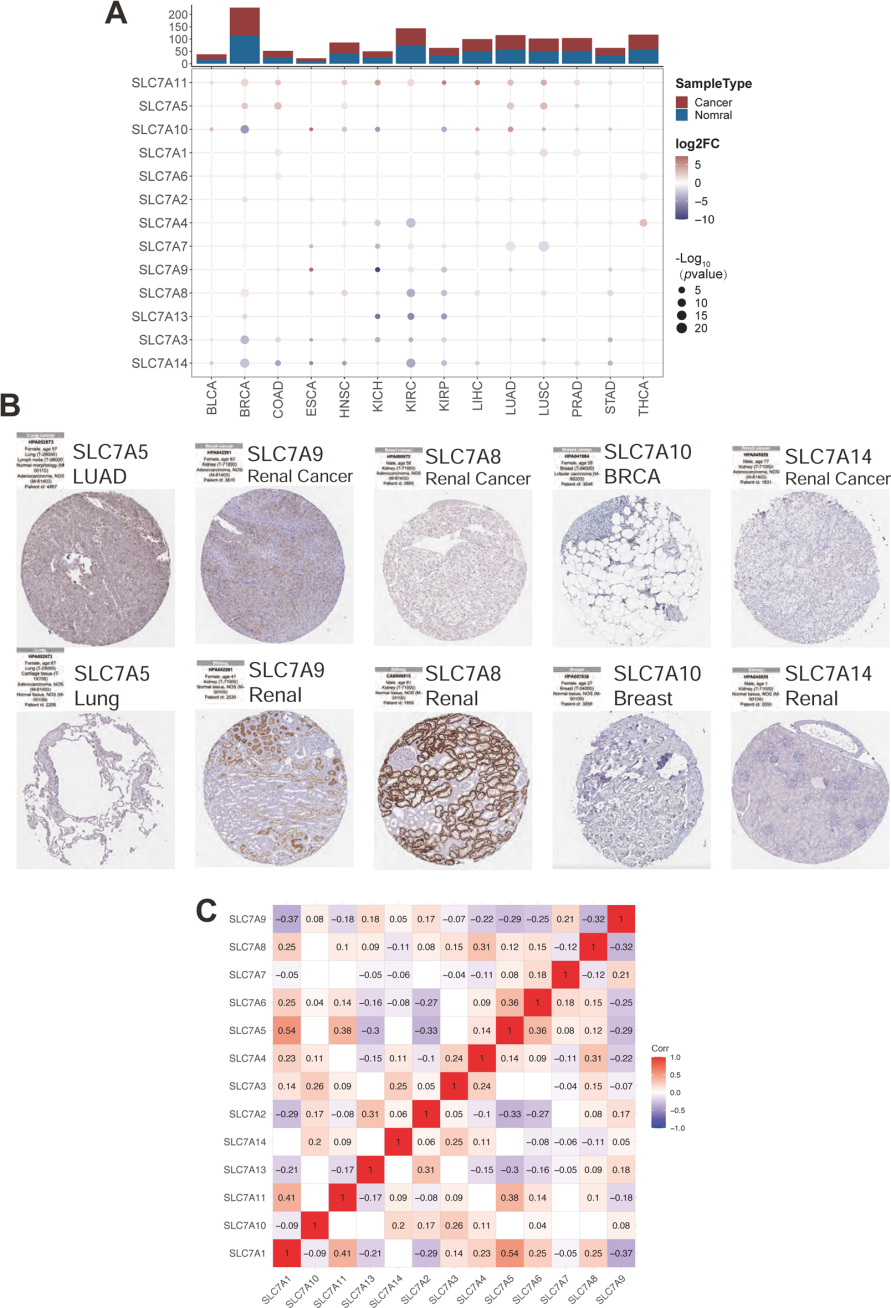
Aberrant Expression Patterns of SLC7 Family Genes in Pan-Cancer. **A** Comparative analysis of the differential expression profiles of the SLC7 family across fourteen TCGA cancer cohorts (each comprising > 10 pairs of tumor-normal samples). **B** Immunohistochemical characterization of the SLC7 family genes utilizing data from the Human Protein Atlas. **C** Pearson’s correlation assessment depicting the interrelationship among SLC7 family genes using TCGA pan-cancer data. Intensity of red coloration indicates the strength of correlation.

In our analysis, we first assessed the differential expression of 14 SLC7 family genes between paired tumor and normal samples across various cancers in the TCGA database (14 out of 33 cancer types). As shown in Fig. 1A, SLC7A11 was significantly upregulated in 12 out of 14 TCGA tumor types, whereas SLC7A14 was notably downregulated in 8 tumor types. These findings were validated using immunohistochemistry data from the Human Protein Atlas (HPA) database (Fig. 1B), which confirmed consistency with RNA-seq-based gene expression analyses. Notably, SLC7A5 and SLC7A9 showed high expression levels in specific tissues, while SLC7A8, SLC7A10, and SLC7A14 were minimally expressed in normal tissues. Pearson’s correlation analysis within the pan-cancer cohort further clarified the relationships between SLC7 family members (Fig. 1C).

**Fig. 2:**
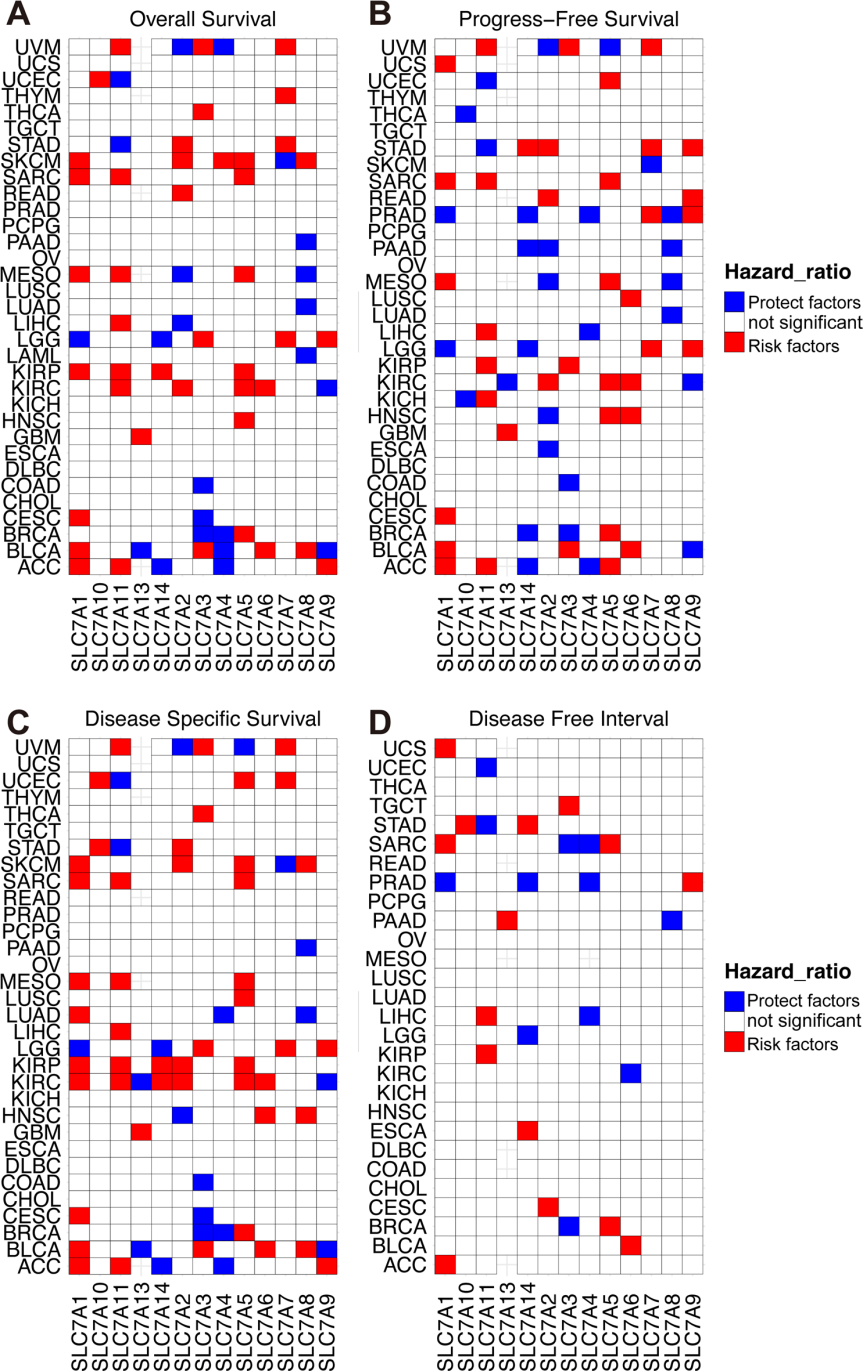
The prognostic value of SLC7 family genes in pan-cancer. A-D Heatmaps illustrating the univariate Cox regression analysis outcomes for SLC7 family genes across various tumor types, assessing their prognostic impact on Overall survival (**A**), Progression-free survival(**B**), Disease-specific survival(**C**), Disease-free interval(**D**).

In the correlation heatmap, SLC7A1 displayed positive correlations with SLC7A11 (*R* = 0.41, *p* < 0.05) and SLC7A5 (*R* = 0.54, *p* < 0.05), and a negative correlation with SLC7A9 (*R*= −0.39, *p* < 0.05) across the pan-cancer cohort. Next, univariate Cox regression analysis was used to investigate the prognostic impact of SLC7 family genes across different cancer types. Higher expressions of SLC7A1, SLC7A11, SLC7A5, and SLC7A7 were associated with poorer outcomes in overall survival (OS, Fig. 2A), progression-free survival (PFS, Fig. 2B), disease-specific survival (DSS, Fig. 2C), and disease-free interval (PFI, Fig. 2D). In contrast, lower expressions of SLC7A8, SLC7A4, SLC7A3, and SLC7A14 were correlated with more favorable prognostic outcomes.

### Effects of gene alterations on slc7 family gene expression

To explore the association between gene alterations in the SLC7 family and gene expression levels, we conducted a comprehensive analysis of the frequencies of somatic mutations, homozygous deletions, heterozygous amplifications, and methylation patterns across the pan-cancer cohort.

**Fig. 3:**
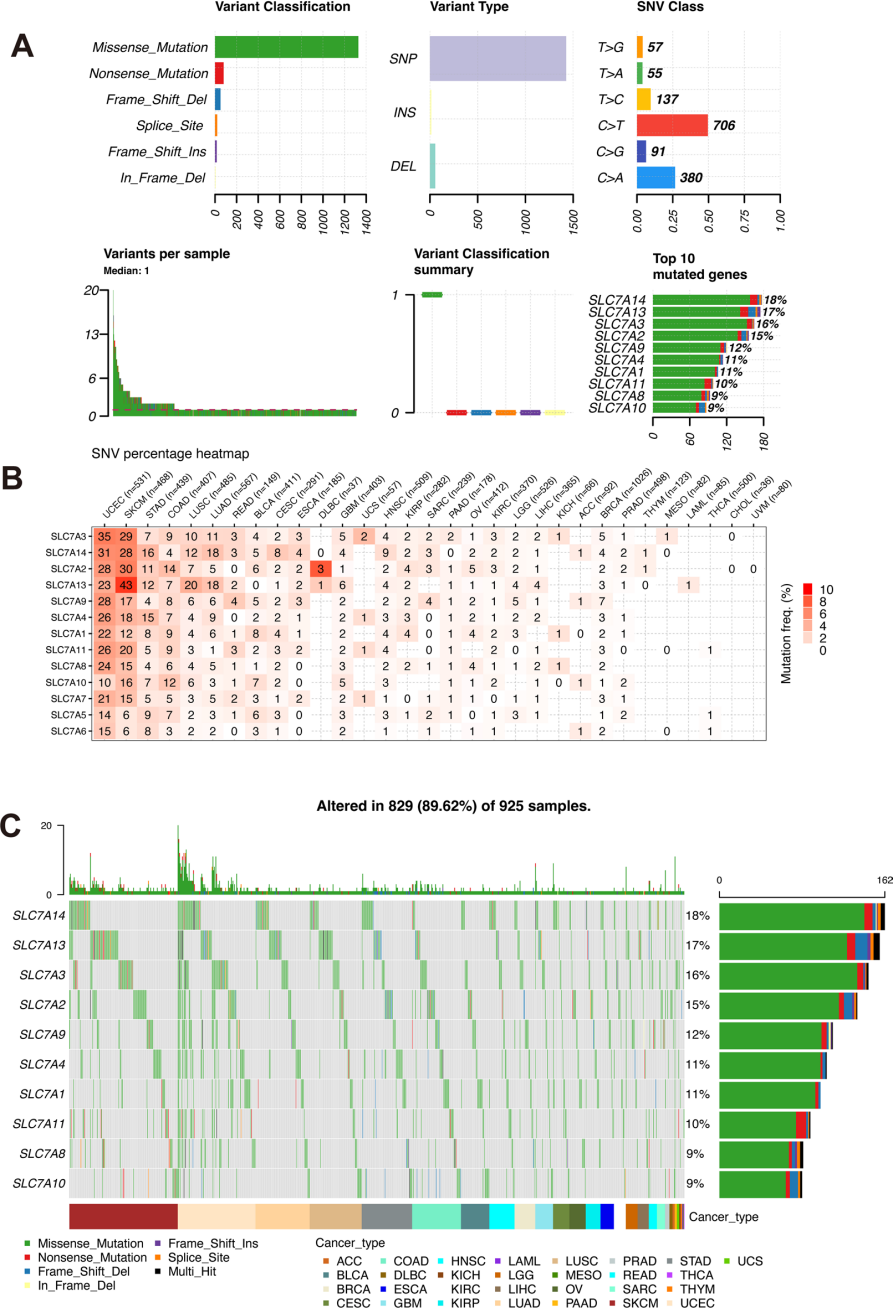
Single-Nucleotide Variant (SNV) Analysis of SLC7 Family Genes. **A** Comprehensive overview of the SNV landscape for SLC7 family genes in pan-cancer. **B** Frequency distribution of deleterious mutations observed in distinct tumor types. **C** Oncoplot representation illustrating the distribution of SNV mutations across the SLC7 family genes within the pan-cancer cohort.

**Fig. 4:**
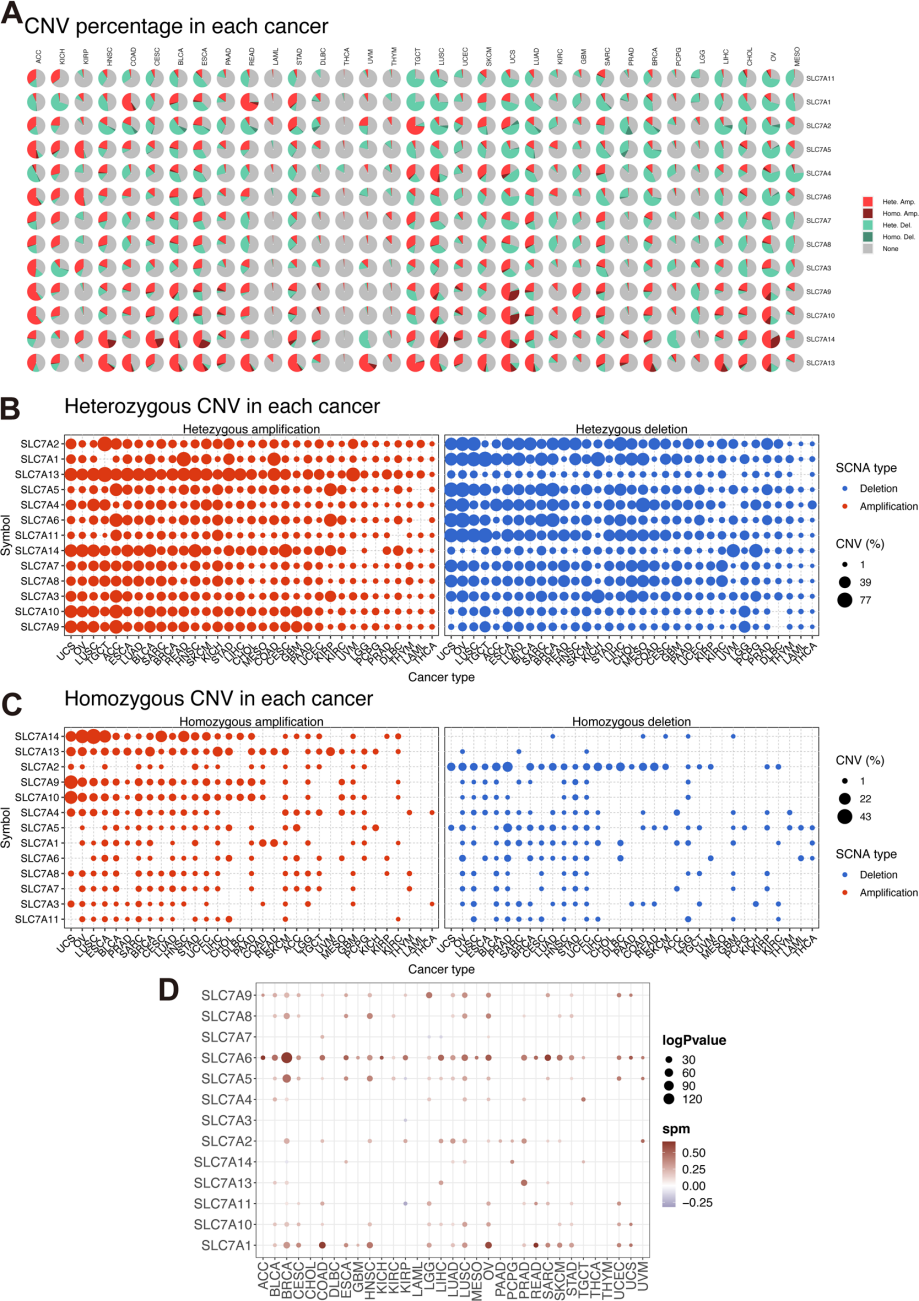
Copy Number Variants (CNV) Analysis of SLC7 Family Genes in Pan-Cancer.** A** Pie chart demonstrating the distribution of CNVs for SLC7 family genes within specific tumor types. **B** Heterozygous CNV profile depicting the proportion of heterozygous alterations (including amplifications and deletions) for each SLC7 family gene across pan-cancer. **C** Homozygous CNV profile illustrating the percentage of homozygous alterations (such as amplifications and deletions) for each SLC7 family gene in the pan-cancer dataset. **D** The correlation between gene expression levels of SLC7 family genes and SLC7 CNVs.

Our analysis revealed a high frequency of deleterious mutations in SLC7A3 in 531 Uterine Corpus Endometrial Carcinoma (UCEC) samples (Fig. 3A-B). Protective genes within the SLC7 family, such as SLC7A13, SLC7A14, and SLC7A3, also exhibited the highest mutation frequencies (16%−18%) among all SLC7 family genes in the pan-cancer cohort (Fig. 3C). Somatic copy number variants (CNVs) and methylation patterns were then examined across the SLC7 family. CNV analyses revealed that heterozygous and homozygous amplifications positively correlated with SLC7 gene expression levels, while deletions showed a negative correlation across cancer types (Fig. 4A-C). Remarkably, in 26 of 33 tumor types, CNV levels of SLC7A6 were significantly correlated with its gene expression levels (Fig. 4D, Table S1).

**Fig. 5:**
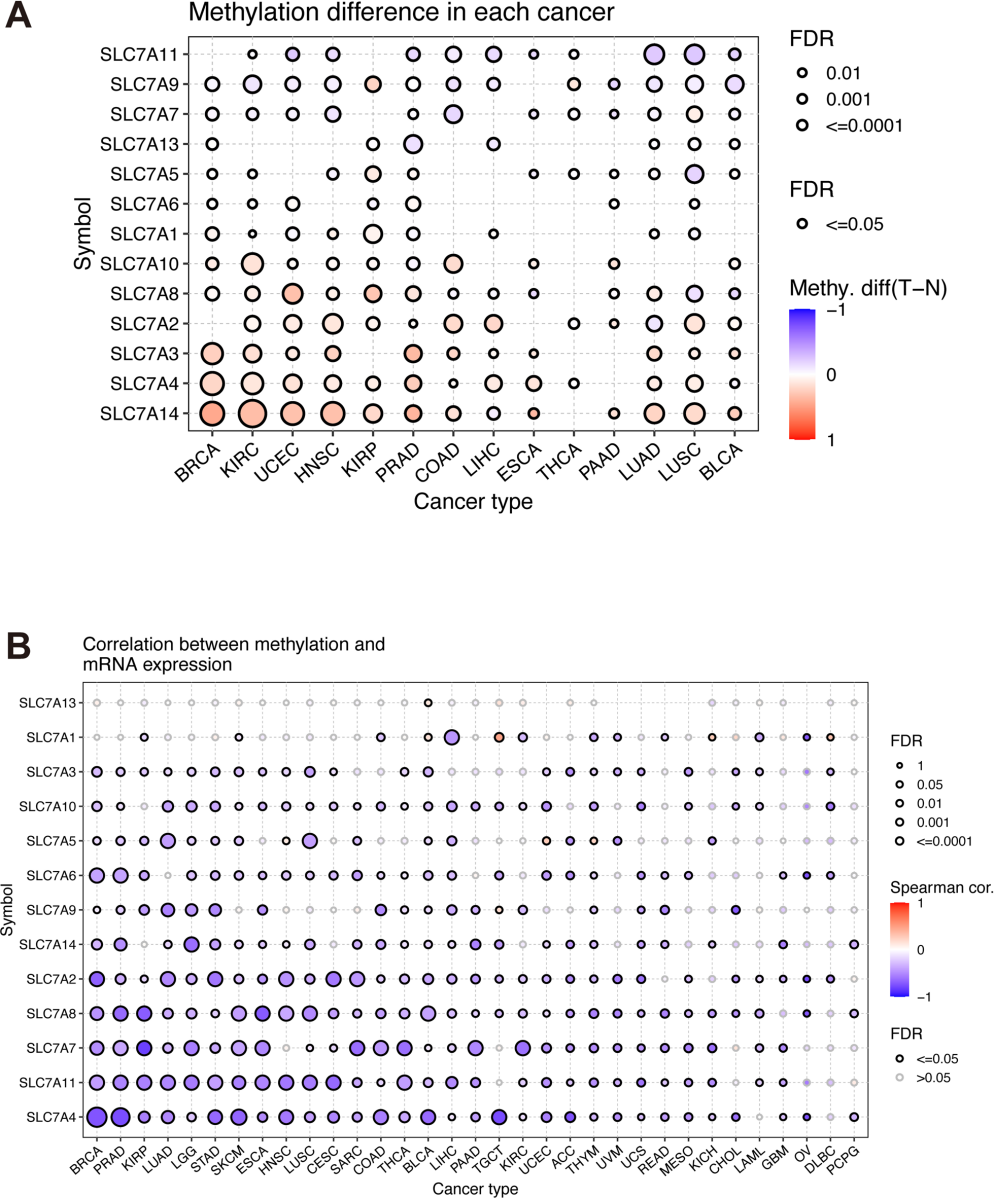
Methylation Patterns of SLC7 Family Genes in Pan-Cancer. **A** Bubble plot providing a comprehensive overview of methylation alterations between tumor and adjacent normal tissues across specific cancer types. The size of each bubble corresponds to the adjusted p-value (FDR), with red dots indicating hypermethylation and blue dots indicating hypomethylation within tumors. Only dots with FDR significance (FDR ≤ 0.05) are displayed. **B** Examination of the correlation between mRNA expression levels of individual SLC7 family genes and aberrant methylation patterns.

Differential methylation analysis indicated significant differences for SLC7A4 between tumor and normal tissues (Fig. 5A, Table S2), with a general negative correlation between methylation levels and gene expression across cancer types (Fig. 5B). SLC7A11 also exhibited negative correlations with both hyper- and hypo-methylation statuses in most cancers (cutoff = 0.25, Table S3).

### Clinical subtype distribution of the SLC7 score

**Fig. 6:**
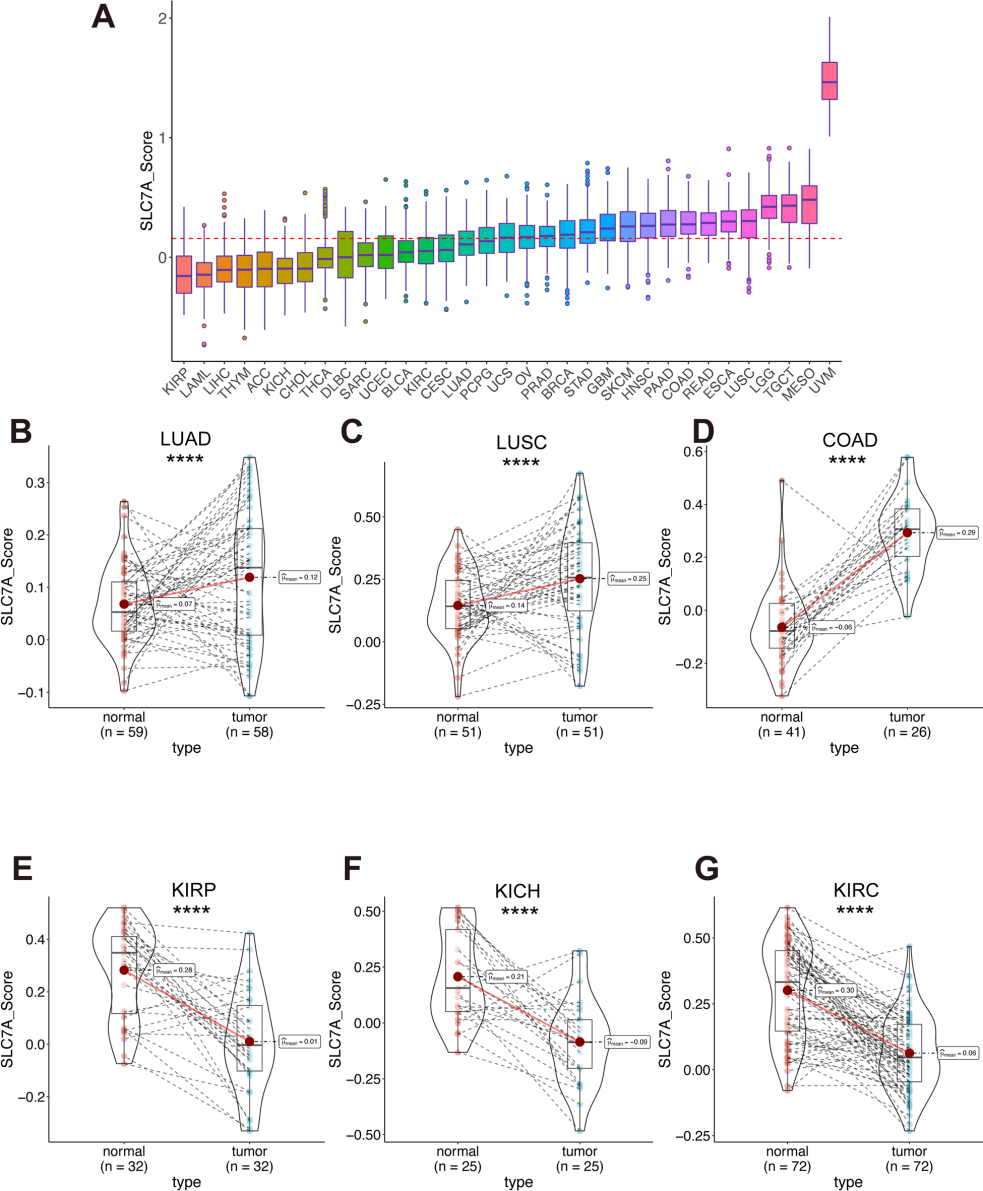
Distribution of SLC7 score in pan-cancer. **A** Overview of the SLC7 score distribution in the TCGA pan-cancer cohort. **B-G** Variations in the distribution of SLC7 scores within paired tumor-normal samples across specific cancer types: LUAD (**B**), LUSC (**C**), COAD (**D**), KIRP (**E**), KICH (**F**) and KIRC (**G**). *p-value < 0.05: **p-value < 0.01: ***p-value < 0.001: ****p-value < 0.0001.

We employed Gene Set Variation Analysis (GSVA) to calculate the SLC7 score across a diverse pan-cancer cohort encompassing 33 cancer types. The SLC7 score was highest in Uveal Melanoma (UVM) and lowest in Kidney Renal Papillary Cell Carcinoma (KIRP) (Fig. 6A). Elevated SLC7 scores were observed in tumor tissues compared to normal tissues in Lung Adenocarcinoma (LUAD), Lung Squamous Cell Carcinoma (LUSC), and Colon Adenocarcinoma (COAD) (Fig. 6B–D), while decreased scores were noted in KIRP, Kidney Chromophobe (KICH), and Kidney Renal Clear Cell Carcinoma (KIRC) (Fig. 6E–G).

**Fig. 7:**
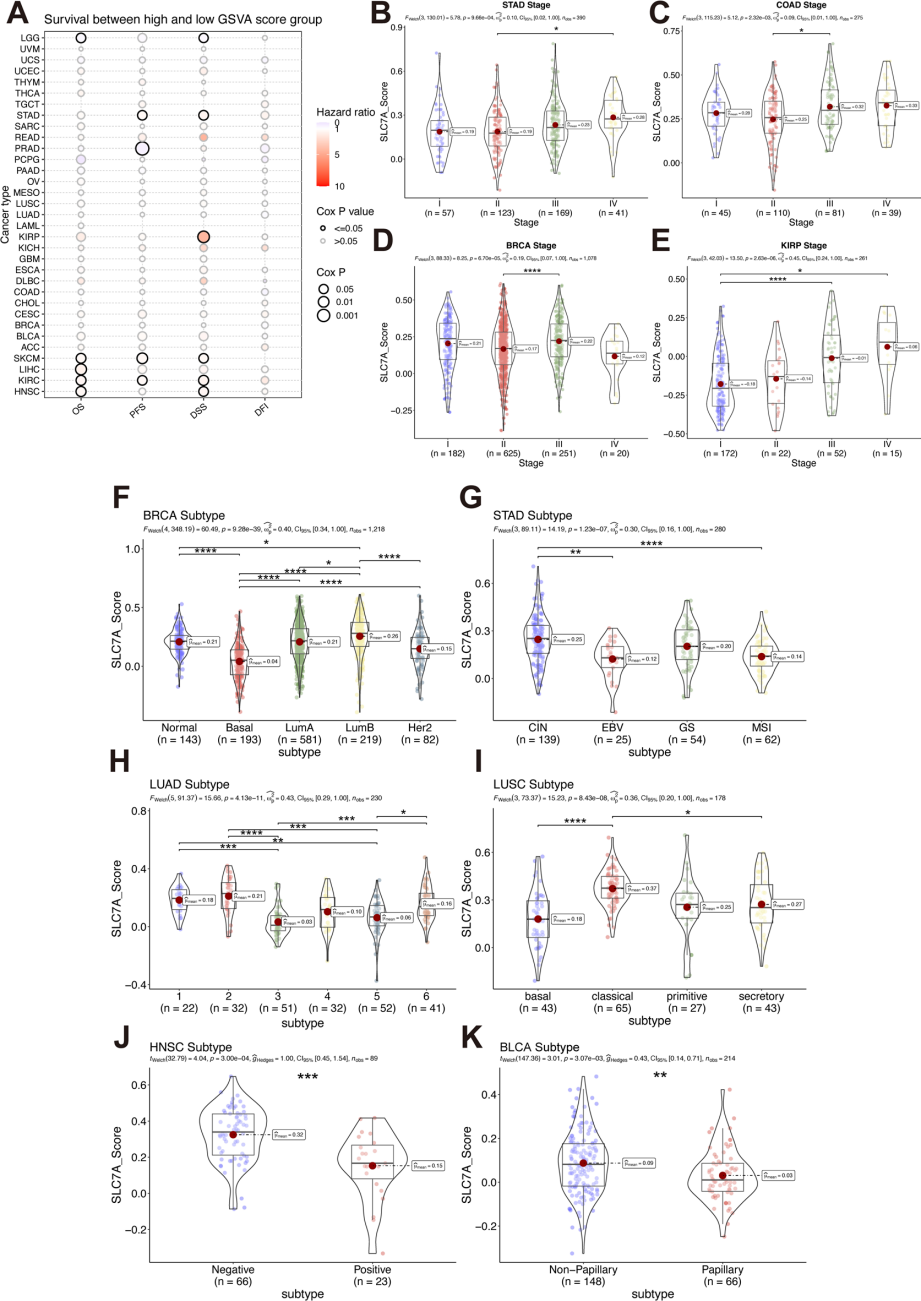
The association of SLC7 score and clinical data.** A** The survival status of SLC7 score distribution in TCGA pan-cancer cohort. **B-E** The stage distribution of SLC7 score in STAD (**b**): COAD (**c**): BRCA (**d**): and KIRP (**e**) cohort. **F-K** The subtype distribution of SLC7 score in tumors: including BRCA (**f**): STAD (**g**): LUAD (**h**): LUSC (**i**): HNSC (**j**): and BLCA (**k**).

In univariate Cox regression analysis, the SLC7 score was a significant risk factor for OS in Skin Cutaneous Melanoma (SKCM), Liver Hepatocellular Carcinoma (LIHC), KIRC, and Head and Neck Squamous Cell Carcinoma (HNSC); for PFS in Stomach Adenocarcinoma (STAD), Prostate Adenocarcinoma (PRAD), SKCM, and KIRC; and for DSS in Low-Grade Glioma (LGG), STAD, KIRP, SKCM, KIRC, and HNSC (Fig. 7A). These findings were further validated by multivariate Cox regression adjusted for stage, age, and gender, confirming the independent prognostic value of the SLC7 score across several cancer types (Figure S1). Analysis across different clinical stages revealed that higher SLC7 scores were associated with advanced tumor grades in Stomach Adenocarcinoma (STAD), Colon Adenocarcinoma (COAD), Breast Invasive Carcinoma (BRCA), and KIRP cohorts (Fig. 7B-E). Additionally, the SLC7 score effectively identified subtypes within cancers such as BRCA, STAD, LUAD, LUSC, HNSC, and Bladder Urothelial Carcinoma (BLCA) (Fig. 7F-K).

### Functional and enrichment analysis of the SLC7 score across cancers

**Fig. 8:**
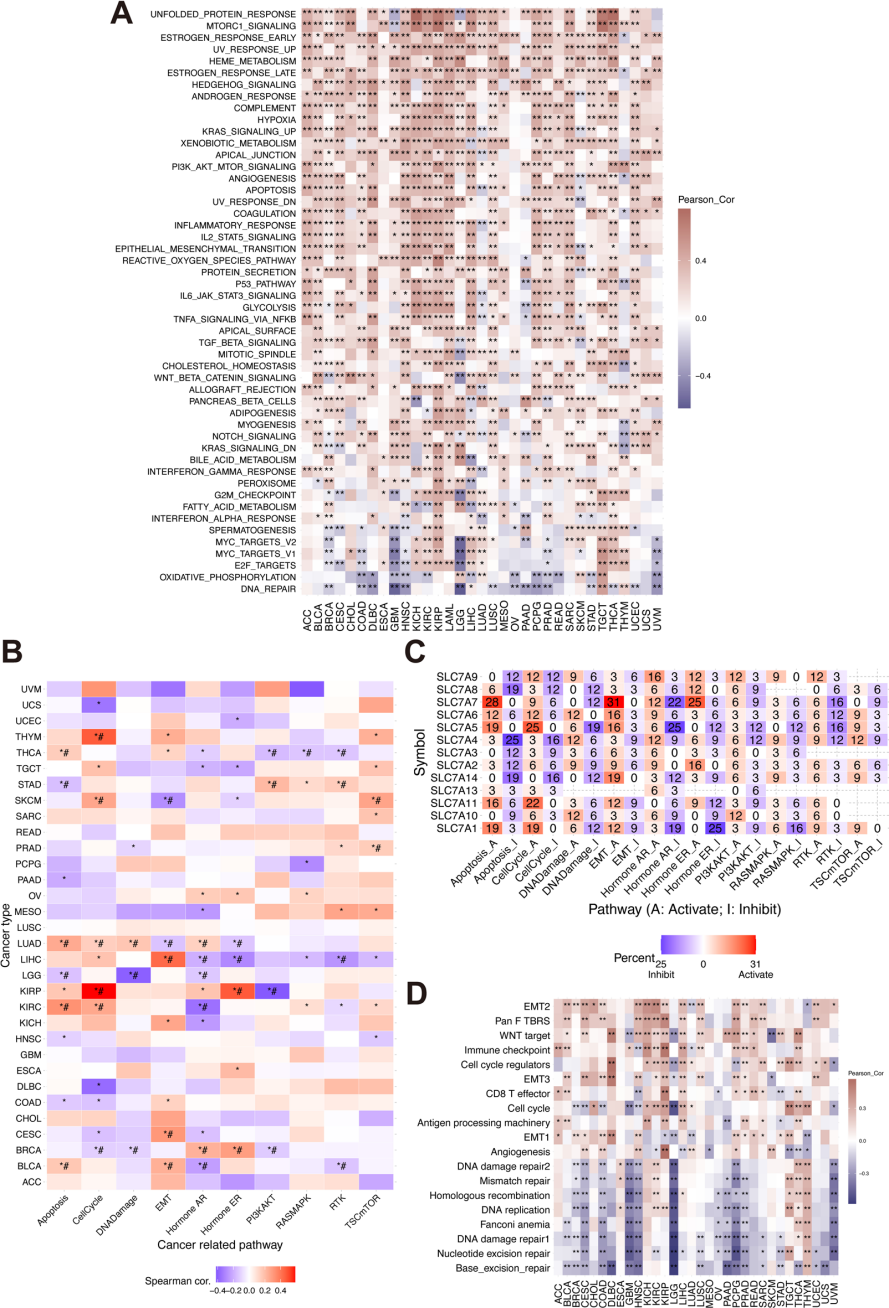
The correlation of SLC7 score and pathway enrichment in Pan-Cancer.** A** Heatmap illustrating the relationship between HALLMARK pathways and SLC7 scores across various cancer types. **B** Heatmap depicting the association between specific pathway activity scores and SLC7 scores in pan-cancer. **C** Exploration of the correlation between specific pathway activity scores and SLC family proteins in pan-cancer. **D** Heatmap representing the correlation between immune-related pathway scores and SLC7 scores in pan-cancer.

To elucidate potential cancer or immune pathways affected by the SLC7 score, we employed GSVA based on 50 HALLMARK pathways. Spearman’s correlation analysis quantified the relationships between the SLC7 score and pathway activity, with the heatmap in Fig. 8A showing correlations across 33 TCGA cancer types. Notably, the SLC7 score was significantly associated with the mTORC1 and Hedgehog pathways.

Using Reverse Phase Protein Array (RPPA) data, we assessed the correlation between SLC7 protein expression and pathway activity (Fig. 8B, Table S4). SLC7A7, SLC7A5, and SLC7A11 frequently correlated with Apoptosis and Cell Cycle pathway activation in over 15 cancer types, while SLC7A4 and SLC7A2 were more commonly associated with pathway inhibition (Fig. 8C). Furthermore, single-sample Gene Set Enrichment Analysis (ssGSEA) indicated significant positive correlations between the SLC7 score and immune-related pathways, including Pan-F TBRS, Immune Checkpoint, and CD8 T Effector signatures across cancers (Fig. 8D, Table S5).

### Interactions between the SLC7 Score and tumor microenvironment

**Fig. 9:**
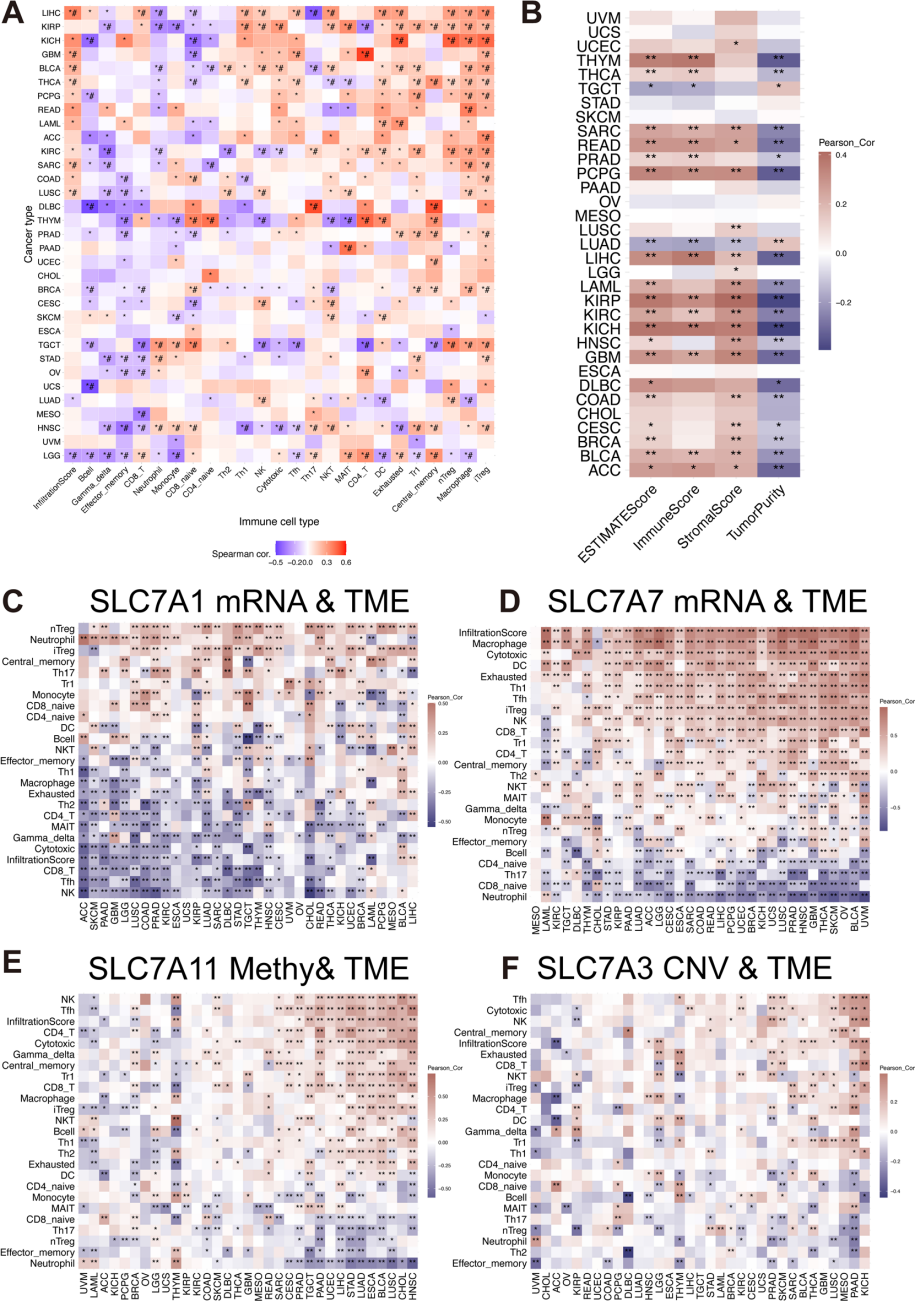
The immune infiltration analyses of pan-cancer.** A** Examination of the association between SLC7 scores and immune cell types utilizing the ImmuCellAI algorithm in pan-cancer. **B** Correlation analysis between SLC7 scores and immune scores based on the ESTIMATE database in pan-cancer. **C-F** Investigation within the pan-cancer cohort focusing on the relationship between immune cell infiltration and mRNA expression of SLC family genes, including: SLC7A1(C), SLC7A7 (D), methylation of SLC7A11 (E) and CNV of SLC7A3 (F).

To explore the interaction between the SLC7 score and the tumor immune microenvironment, we applied ImmuCellAI to assess immune cell infiltrations within the pan-cancer cohort. Higher SLC7 scores correlated positively with Treg and Macrophage populations and negatively with B cells and effector memory cells (Fig. 9A). Additionally, the SLC7 score positively correlated with stromal and immune content scores, while negatively correlating with tumor purity across cancers (Fig. 9B).

Further analysis identified SLC7A1 as the gene most negatively correlated with the Tumor Microenvironment (TME) (Fig. 9C), while SLC7A7 showed the highest positive correlation with the TME (Fig. 9D). Methylation levels of SLC7A11 positively correlated with NK cells, Tfh cells, and CD4 T cells across multiple cancers (Fig. 9E). CNV levels of SLC7A3 positively correlated with Tfh, NK, and CD8 T cells in Kidney Chromophobe (KICH) and Pancreatic Adenocarcinoma (PAAD) (Fig. 9F).

**Fig. 10:**
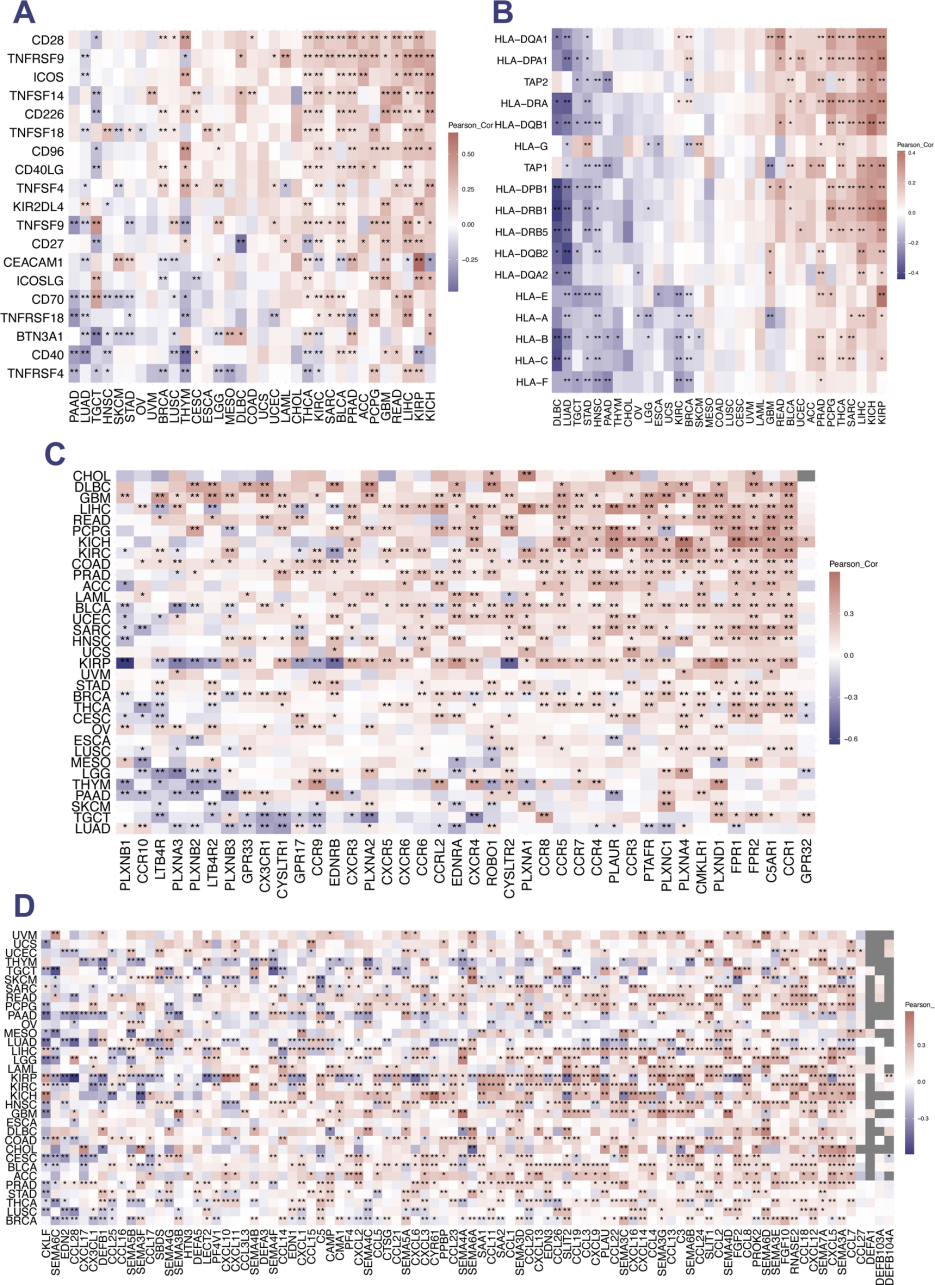
Association of SLC7 Score with Immune-Related Factors in Pan-Cancer.** A** Investigation into the correlation between SLC7 scores and immune-activated genes across diverse cancer types. **B** Assessment of the association between SLC7 scores and Major Histocompatibility Complex (MHC) genes in pan-cancer. **C** Exploration of the relationship between SLC7 scores and chemokine receptors in the context of various cancer types. **D** Examination of the correlation between SLC7 scores and chemokines across different cancer types.

Significant correlations were also found between the SLC7 score and immune-activated genes(Fig. 10A), MHC(Fig. 10B), chemokine receptors(Fig. 10C) and chemokines(Fig. 10D), suggesting that elevated SLC7 scores may enhance immune cell infiltration, with implications for chemotherapy and immunotherapy responsiveness.

### Association between SLC7 family genes and drug response

**Fig. 11:**
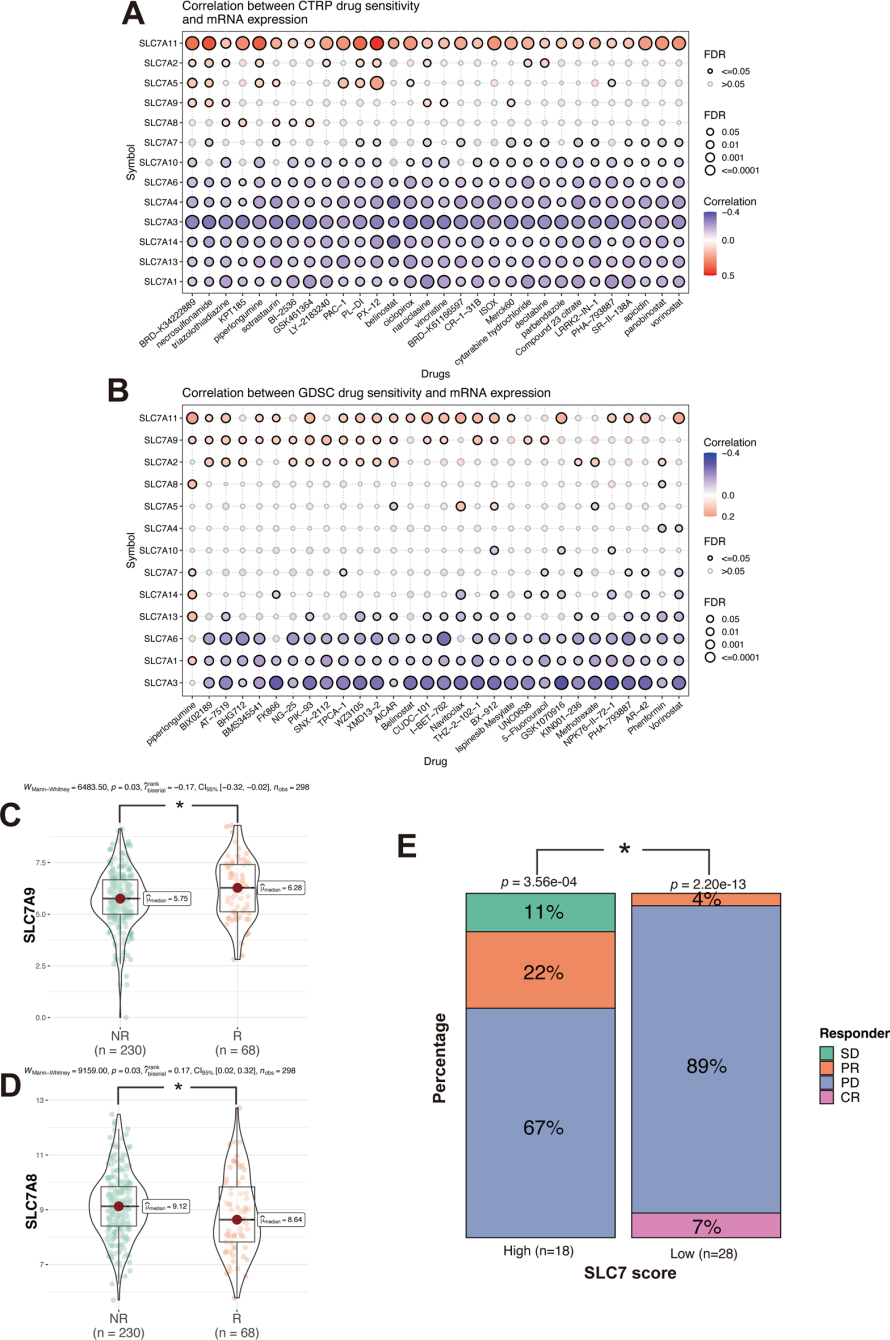
Correlation between SLC7 Scores and Drug Sensitivity/Immunotherapy Response.** A-B** Examination of the association between mRNA expression levels of SLC7 family genes and drug sensitivity values using the CTRP (A) and GDSC (B) databases. **C-E** Analysis within IMvigor210 cohorts focusing on the percentage of patients responsive to Atezolizumab treatment in high and low groups based on: SLC7 score (C), SLC7A8 (D) and SLC7A9 (E). SD: stable disease: PD: progressive disease: PR: partial response: CR: complete response: NR: SD + PD: R: CR + PR.

To investigate the SLC7 score’s relevance to drug sensitivity, we analyzed the correlation between half-maximal inhibitory concentration (IC50) values and SLC7 family gene expression levels using data from the CTRP and GDSC databases. In CTRP, higher expression of SLC7A11 correlated positively with IC50 values, whereas other SLC7 genes (e.g., SLC7A1, SLC7A13, SLC7A14) showed negative correlations with IC50 values (Fig. 11A).

In the GDSC dataset, similar trends were observed (Fig. 11B). Additionally, within the Bladder Urothelial Carcinoma (BLCA) cohort, non-responders to atezolizumab treatment exhibited higher SLC7A8 and lower SLC7A9 expression (Figs. 11C, D). Patients with higher SLC7 scores also showed partial or stable responses to PD-L1 inhibition, while those with lower scores were more likely to achieve complete response or experience progressive disease (Fig. 11E).

### SLC7A5 is required for KRAS-driven proliferation and invasion of cancer cells

Given the strong association between SLC7 family members expression and poor prognosis in multiple cancers, we next sought to experimentally validate the expression patterns and functional roles of key SLC7 family members in tumor cell lines.

**Fig. 12:**
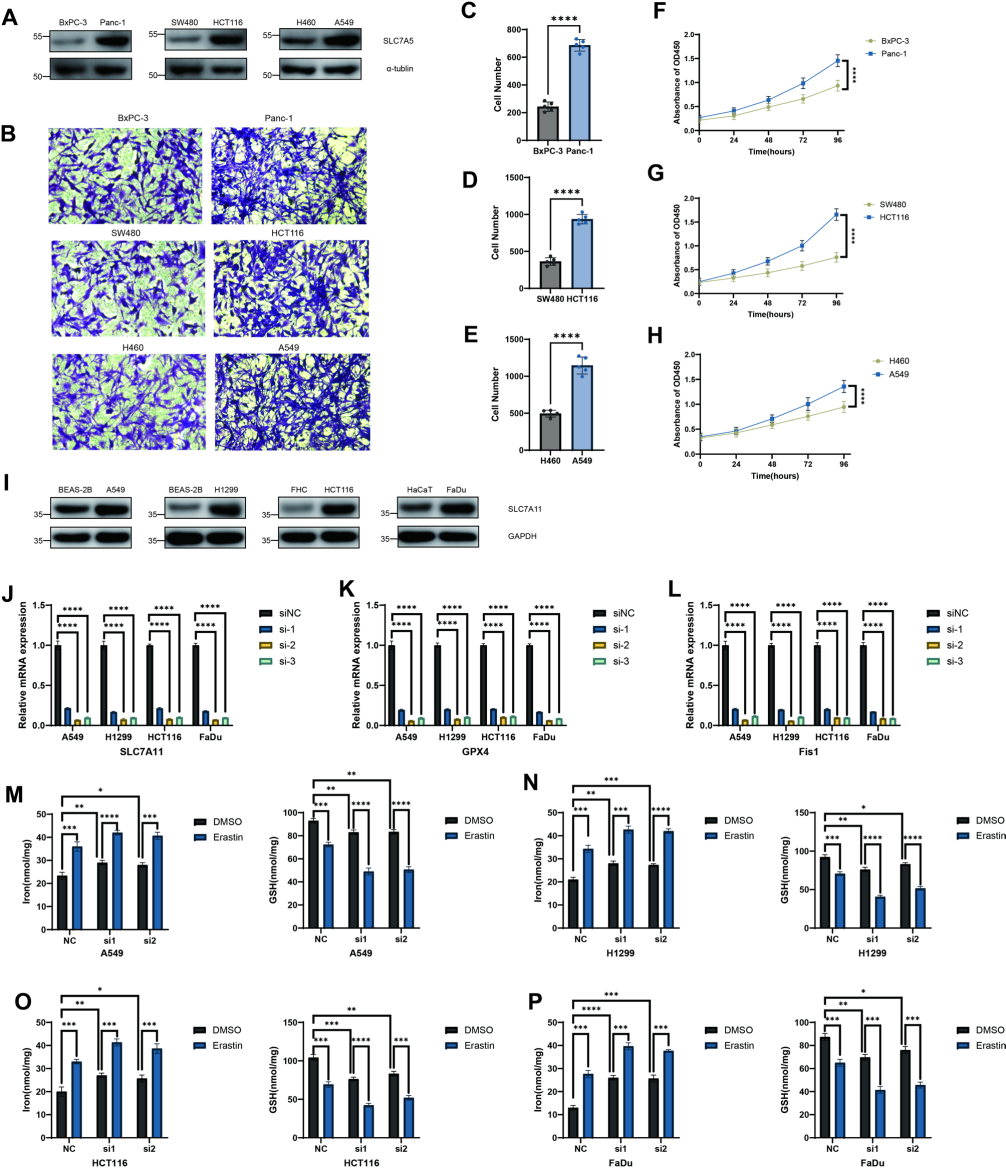
SLC7A5 promotes KRAS-driven tumor aggressiveness and SLC7A11 protects cancer cells from ferroptosis.** A** Western blot analysis showing elevated SLC7A5 protein levels in KRAS-mutant (Panc-1, HCT116, A549) compared with KRAS-wildtype (BxPC-3, SW480, H460) cancer cell lines. **B–E** Representative images and quantification of Transwell invasion assays demonstrating enhanced invasive abilities of KRAS-mutant cells. **F–H** Cell proliferation curves assessed by CCK-8 assays showing accelerated growth in KRAS-mutant lines. **I** Western blot showing increased SLC7A11 expression in tumor cell lines (A549, H1299, HCT116, FaDu) compared with normal counterparts (BEAS-2B, FHC, HaCaT). **J–L** qRT–PCR analysis confirming efficient SLC7A11 knockdown and subsequent downregulation of GPX4 and Fis1 in indicated cancer cell lines. **M–P** Measurement of intracellular iron and GSH levels showing that SLC7A11 depletion enhances erastin-induced ferroptosis. Data represent mean ± SD from three independent experiments (**P < 0.01, ***P < 0.001, ****P < 0.0001).

We first examined its expression profile across a panel of cancer cell lines with different KRAS mutation statuses to determine whether SLC7A5 is involved in KRAS-driven tumor progression. Western blot analysis revealed that SLC7A5 protein levels were markedly elevated in KRAS-mutant cell lines, including pancreatic cancer (Panc-1), colorectal cancer (HCT116), lung adenocarcinoma (A549), compared with KRAS-wildtype counterparts such as BxPC-3, SW480, and H460 (Fig. 12A). This differential expression pattern suggested a potential link between KRAS oncogenic signaling and SLC7A5 upregulation.

To further assess the functional relevance of SLC7A5 in KRAS-driven malignancy, we performed Transwell invasion assays. KRAS-mutant cells displayed significantly enhanced invasive abilities compared with KRAS-wildtype cells, consistent with their higher SLC7A5 expression (Fig. 12B-E). In parallel, cell proliferation curves demonstrated that KRAS-mutant cells (Panc-1, HCT116, A549) exhibited a faster growth rate than wildtype lines (BxPC-3, SW480, H460) (Fig. 12F–H). Together, these results indicate that SLC7A5 is positively associated with KRAS mutation status and may serve as a downstream effector facilitating KRAS-mediated metabolic reprogramming, thereby promoting tumor cell proliferation and invasion.

### SLC7A11 protects cancer cells from ferroptosis through maintaining redox homeostasis

Given the metabolic interplay between amino acid transport and redox regulation, we next investigated the role of SLC7A11 in ferroptosis resistance. Western blotting revealed that SLC7A11 expression was substantially higher in various cancer cell lines, including colorectal cancer (HCT116), lung adenocarcinoma (A549), non-small cell lung cancer (H1299), and head and neck squamous carcinoma (FaDu), compared with their corresponding normal counterparts (BEAS-2B, FHC, and HaCaT) (Fig. 12I), implying that tumor cells may depend on SLC7A11 for survival under oxidative stress.

To elucidate its functional role, we silenced SLC7A11 expression using three independent siRNAs. Quantitative PCR confirmed efficient knockdown in all tested cancer cell lines (Fig. 12J). Notably, depletion of SLC7A11 led to a pronounced reduction in the expression of key ferroptosis-related regulators, including GPX4 and Fis1 (Fig. 12K–L), indicating impaired antioxidant capacity. Functionally, treatment with the ferroptosis inducer Erastin significantly increased the extent of ferroptosis and decreased GSH levels following SLC7A11 knockdown. (Fig. 12M–P).

Collectively, these data demonstrate that SLC7A11 acts as a crucial suppressor of ferroptosis by sustaining intracellular cystine uptake and maintaining redox balance. Its overexpression in cancer cells provides a metabolic advantage that confers resistance to oxidative stress and ferroptotic cell death, highlighting SLC7A11 as a potential therapeutic target for overcoming ferroptosis resistance in tumors.

## Discussion

Amino acid (AA) metabolism and the interplay between diverse cell types drive metabolic reprogramming within the tumor immune microenvironment (TIME), posing significant challenges for therapeutic strategies in cancer patients^1^. Among AA transporters, the SLC7 family has emerged as a central component, influencing tumor metabolism dynamics^3^. Yet, the roles of SLC7 family members as either tumor-promoting or suppressing factors remain incompletely characterized across cancer types.

In our study, we systematically evaluated the differential expression and prognostic relevance of 14 SLC7 family genes using the TCGA database. Key findings highlight the roles of specific SLC7 genes, notably SLC7A1, SLC7A5, SLC7A7, and SLC7A11, as prognostic indicators in multiple cancers (Figs. 1 and 2). These findings resonate with prior studies^9,13,31,32^. For instance, SLC7A11 upregulation is known to drive tumor proliferation by inhibiting ferroptosis, suggesting that high SLC7A11 levels may mark metabolic vulnerabilities for targeted therapies^31,32^. SLC7A5 is essential for the proliferation of KRAS-mutant colorectal cancer^9^. The expression of SLC7A7 was upregulated and correlated with immune infiltrates in lung cancer^13^.

To further explore the oncogenic and suppressive potentials of SLC7 family genes, we analyzed their genomic and epigenetic alterations, including SNVs, CNVs, and methylation patterns. Results indicate that deleterious mutations and CNV increases in genes such as SLC7A6 and SLC7A14 contribute to tumor progression. AAs are essential for both tumor and immune cells, as AA availability and metabolism are pivotal for T cell activation and function^26,33^. Tumor cells utilize AAs not only for tumorigenesis and proliferation^34^, but also to enable tumor immune evasion^35,36^. Using the GSVA algorithm, we constructed an SLC7 score across a pan-cancer cohort, observing elevated scores in tumor tissues, including UVM, MESO, TGCT, LGG, and LUSC, and lower scores in KIRP, LIHC, THYM, and KICH. Additionally, tumors with high SLC7 scores, such as LIHC, KIRC, LGG, SKCM, and HNSC, tend to correlate with poorer prognoses. Enrichment analyses revealed a positive correlation between the SLC7 score and multiple immune pathways, including interferon-gamma response, IL6/JAK/STAT3 signaling, immune checkpoints, and inflammatory responses, all highly relevant to TIME^37–39^. This was further supported by correlations between the SLC7 score and various immune cell populations. Notably, among SLC7 members, SLC7A11 promotes tumor progression by importing cysteine to support glutathione synthesis, thereby inhibiting ferroptosis and enhancing oxidative stress resistance^40^. Meanwhile, SLC7A5 contributes to tumorigenesis by transporting leucine to activate the mTOR pathway, fostering protein synthesis and cell proliferation^41^. Both transporters may also facilitate an immunosuppressive TIME through mechanisms such as nutrient competition with immune cells. These findings provide a reference for further investigation into the specific mechanisms by which alterations in the expression, copy number, and methylation of other genes in the SLC7 family contribute to poor prognosis and immunosuppression.

Moreover, the SLC7 score was associated with immune features such as Pan-F TBRs, antigen processing machinery, and immune checkpoint markers in specific tumors. Procaccini et al. demonstrated SLC7A11’s role in Treg cell proliferation^42^, while it is also a negative regulator of efferocytosis, contributing to inflammatory wound healing in diabetic models^43^. Additionally, we showed a positive correlation between the SLC7 score and immune-activating genes, chemokines, receptors, and MHC genes in numerous tumor types, supporting studies indicating that SLC7A5 is essential for activated CD8 + T cell responses^24,25^. Similarly, SLC7A1-4 were implicated in various immune functions by importing arginine, which is metabolized into several active molecules^27^. In conclusion, our findings suggest that tumors with high SLC7 scores are enriched in cancer-associated signaling and immune cell infiltration, which could influence therapeutic strategy selection. We utilized databases to predict drug response types for patients with high SLC7 scores and found that SLC7A8 and SLC7A9 may serve as predictive markers for immunotherapy responsiveness.

In this study, we identified distinct yet complementary oncogenic roles of two amino acid transporters, SLC7A5 and SLC7A11, in regulating tumor growth and survival. Our findings demonstrate that SLC7A5 is closely associated with KRAS-driven proliferation and invasion, while SLC7A11 confers resistance to ferroptosis by maintaining redox homeostasis. These results highlight the metabolic plasticity of cancer cells, which rely on coordinated amino acid transport and utilization to support malignant progression.

KRAS mutations are well recognized as key oncogenic drivers in multiple solid tumors, particularly pancreatic, colorectal, and lung cancers^44^. However, direct targeting of KRAS has long been challenging due to its structural characteristics and functional redundancy. Our data reveal that KRAS-mutant cancer cells exhibit markedly elevated SLC7A5 expression, suggesting that SLC7A5 may act as a critical downstream effector of KRAS signaling. SLC7A5, also known as LAT1, is a high-affinity transporter for essential neutral amino acids, particularly leucine, which serves as a key activator of the mTORC1 pathway^45^. Therefore, enhanced SLC7A5 expression in KRAS-mutant cells likely promotes amino acid influx, stimulates mTORC1 activity, and thereby drives biosynthetic metabolism and uncontrolled proliferation. The observation that KRAS-mutant cells displayed both increased invasive potential and accelerated growth supports the notion that SLC7A5 is an indispensable metabolic node linking KRAS signaling to nutrient sensing and anabolic growth. Targeting SLC7A5 may thus represent an alternative approach to suppress KRAS-driven tumorigenesis.

In parallel, we found that SLC7A11 plays a pivotal role in protecting cancer cells from ferroptotic cell death. As a component of the cystine/glutamate antiporter system Xc⁻, SLC7A11 mediates cystine uptake for glutathione synthesis, thereby preserving intracellular redox balance^46^. Our data show that silencing SLC7A11 downregulates the expression of GPX4 and Fis1, two essential regulators that detoxify lipid peroxides and maintain iron homeostasis. Functionally, SLC7A11 depletion sensitized tumor cells to Erastin-induced ferroptosis, resulting in elevated lipid peroxidation and cell death. These findings are consistent with previous reports that upregulated SLC7A11 expression enables tumor cells to withstand oxidative stress and contributes to therapy resistance. From a therapeutic perspective, combining SLC7A11 inhibition with ferroptosis inducers may provide a synergistic strategy for eliminating ferroptosis-resistant cancer cells.

Despite the comprehensive bioinformatic analysis of SLC7 family genes, several limitations should be noted. Our findings, primarily derived from computational analyses, require validation in animal models and patient-derived samples to confirm the proposed functions of SLC7 genes. Moreover, although significant correlations between SLC7 alterations and immune evasion or therapy resistance were observed, causal mechanisms remain to be clarified. The study also offers a static snapshot, lacking temporal and spatial insights into SLC7 dynamics during tumor progression or treatment. Future longitudinal and mechanistic investigations will be essential to address these limitations and uncover novel pathways regulated by SLC7 transporters.

## Conclusion

The study underscores a compelling link between elevated SLC7 scores and the pan-cancer tumor immune microenvironment (TIME). Notably, the SLC7 score, along with specific SLC gene family members, has emerged as a promising biomarker for evaluating immunotherapy efficacy across diverse tumor types. These findings position SLC7 family genes as valuable prognostic indicators and potential therapeutic targets, highlighting their innovative role in advancing oncological treatment strategies.

## Supplementary Information

Below is the link to the electronic supplementary material.

Supplementary Material 1

Supplementary Material 2

Supplementary Material 3

Supplementary Material 4

Supplementary Material 5

Supplementary Material 6

Supplementary Material 7

## Data Availability

All data on the measured ecosystem variables indicating ecosystem functions that support the findings of this study are included within this paper and its Supplementary Information files.
