# Supplementary figures and images for: Integrative pan-cancer analysis reveals the prognostic and immunological significance of SLC7 family cationic amino acid transporters
Source: Sci Rep. 2026 Jan 8;16:4500. doi: 10.1038/s41598-025-34723-8 (PMC12865201; doi:10.1038/s41598-025-34723-8)

Multivariate Cox Regression of SLC7A Score  
Adjusted by Stage, Age and Gender

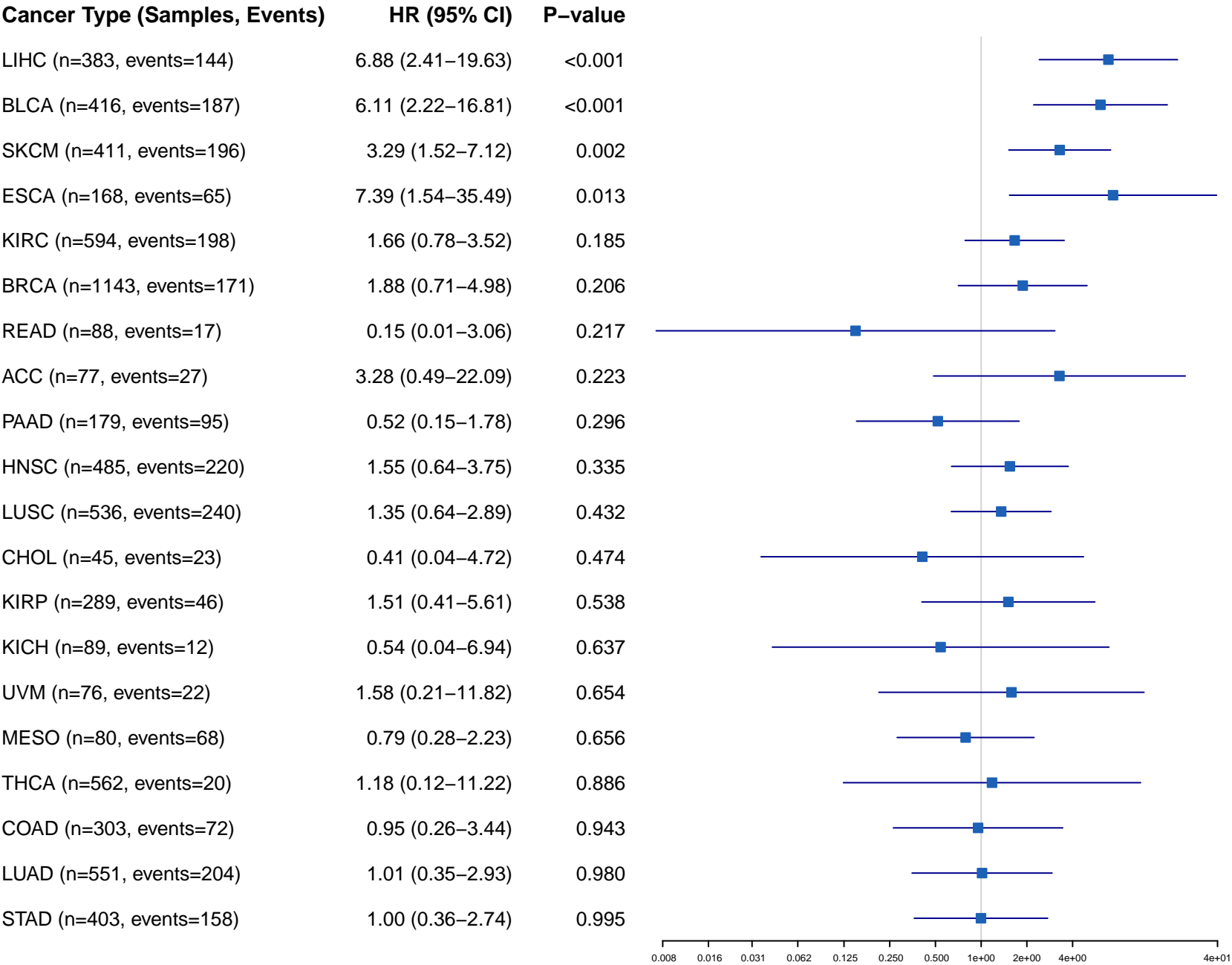

Supplement: Supplementary file 6 — Supplementary Material 6 [file 41598_2025_34723_MOESM6_ESM.pdf]

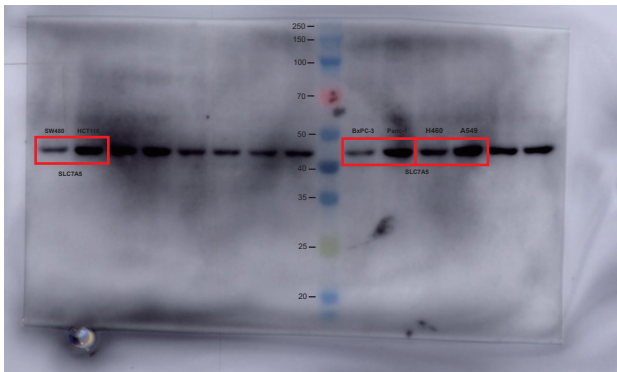

EMulticolor Prestained Protein Ladder  
Pizyme WJ103

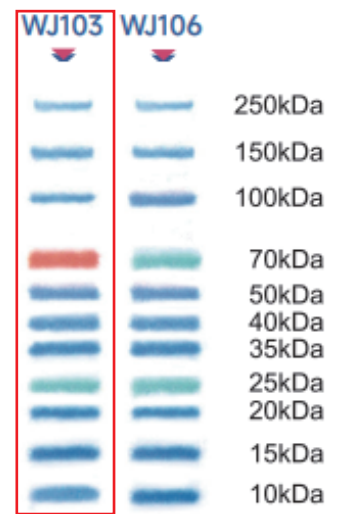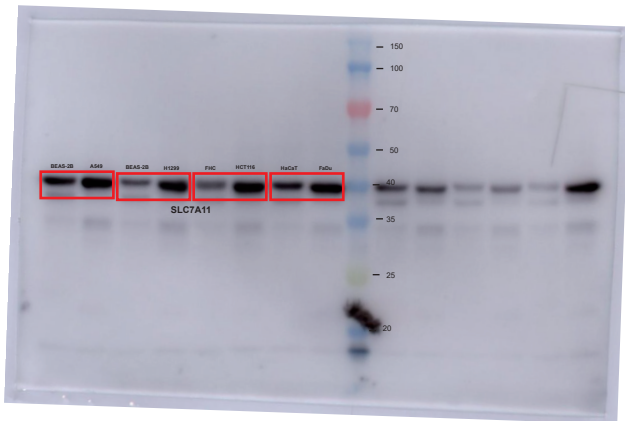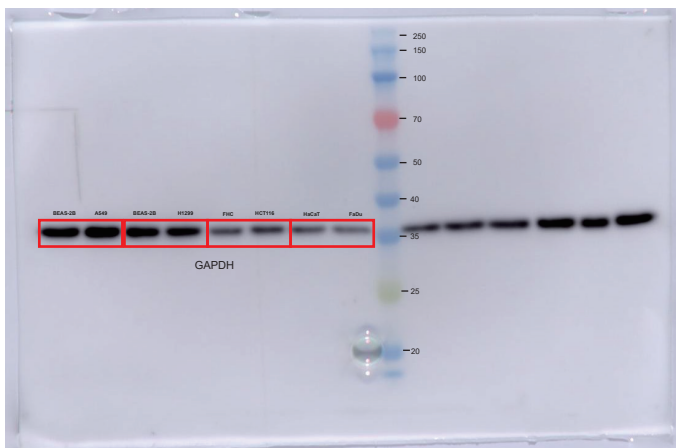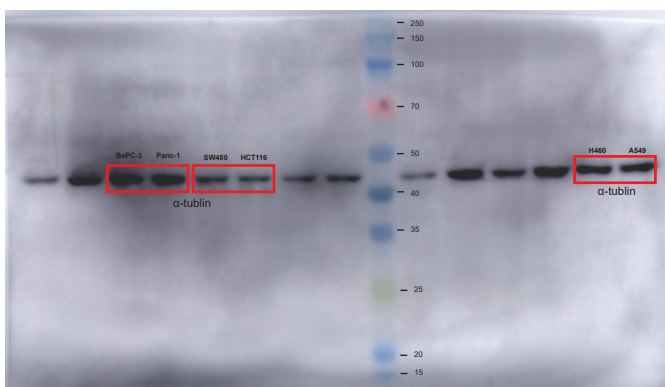

Supplement: Supplementary file 7 — Supplementary Material 7 [file 41598_2025_34723_MOESM7_ESM.pdf]
